# Dynamic Mechanical Behavior of Graphene Oxide Functionalized Curaua Fiber-Reinforced Epoxy Composites: A Brief Report

**DOI:** 10.3390/polym13111897

**Published:** 2021-06-07

**Authors:** Ulisses Oliveira Costa, Lucio Fabio Cassiano Nascimento, Wendell Bruno Almeida Bezerra, Vinícius de Oliveira Aguiar, Artur Camposo Pereira, Sergio Neves Monteiro, Wagner Anacleto Pinheiro

**Affiliations:** 1 Composite Materials Group, Department of Materials Science, Military Institute of Engineering, IME, Rio de Janeiro 22290-270, Brazil; LUCIO_COPPE@yahoo.com.br (L.F.C.N.); wendellbez@gmail.com (W.B.A.B.); camposo.artur@gmail.com (A.C.P.); snevesmonteiro@gmail.com (S.N.M.); waganacleto@gmail.com (W.A.P.); 2 Department of Polymer Science and Technology, Institute of Macromolecules Professor Eloisa Mano, IMA, Rio de Janeiro 21941-598, Brazil; nviny815@gmail.com

**Keywords:** DMA, graphene oxide, curaua fiber, epoxy composite

## Abstract

The coating of natural fiber by graphene oxide (GO) has, over, this past decade, attracted increasing attention as an effective way to improve the adhesion to polymer matrices and enhance the composite properties. In particular, the GO-functionalized 30 vol% curaua fiber (*Ananas Erectifolius*) reinforcing epoxy composite was found to display superior tensile and thermogravimetric properties as well as higher fiber/matrix interfacial shear strength. In this brief report, dynamic mechanical analysis (DMA) was conducted in up to 50 vol% GO-functionalized curaua fiber reinforced epoxy matrix (EM) composites. The objective was not only to extend the amount incorporated but also for the first time investigate the composite viscoelastic behavior. The GO functionalization of curaua fibers (GOCF) improved the DMA storage (E′) and loss (E″) modulus compared to the non-functionalized fiber composites. Values at 30 °C of both E′ (13.44 GPa) and E″ (0.67 GPa) for 50 vol% GO-functionalized curaua fiber reinforced epoxy matrix composites (50GOCF/EM) were substantially higher than those of 20 GOCF/EM with E′ (7.08 GPa) and E″ (0.22 GPa) as well as non-functionalized 50CF/EM with E′ (11.04 GPa) and E″ (0.45 GPa). All these results are above the neat epoxy previously reported values of E′ (3.86 GPa) and E″ (0.09 GPa). As for the tangent delta, the parameters associated with damping factor and glass transition temperature were not found to be significantly changed by GO functionalization, but decreased with respect to the neat epoxy due to chain mobility restriction.

## 1. Introduction

The past decade has witnessed an exponential growth in research works on the expected next-generation classes of natural fiber polymer composites (NFPC) with special characteristics for functional and advanced applications. One class refers to sustainable biocomposites with biodegradable matrices [1,2]. A second class of nanocomposites display significant advantages when compared to conventional composites [3,4]. Incorporation of graphene and graphene-based materials, either separately as in both fiber and matrix, constitutes another successful class of NFPC with enhanced properties [5]. Plain graphene with a different number of layers as in monolayer pristine graphene (PG), few-layers graphene (FLG), and multilayer graphene (MGL) as well as graphene-based materials such as exfoliated nanoplatelet (GNP), flakes (GF), nanoparticles (NG), carbon nanotubes (CNT), oxide (GO), and reduced graphene oxide (rGO) are contributing to substantially improve the performance of polymer composites. For example, as nanopowder, graphene enhances the thermo-mechanical properties of polymer nanocomposites [6]. It is also worth mentioning that the development of a novel material based on graphene layer, the multi-walled carbon nanotube (MWCNT), has been reported to be an efficient reinforcement for polymer composites applicable as high conductive industrial components [7,8,9]. In particular, the interest of the present work was to focus to the advantages of GO. Indeed, very small amounts of GO (<1 wt.%) either coating the natural fiber or incorporated in the polymer matrix were found to significantly improve the NFPC properties [9,10,11,12,13,14,15,16,17,18]. Moreover, GO has been used on cotton fabric as a flexible strain sensor [19] and as an amphiphilic coating for cotton textiles [20].

Among the properties enhanced by GO addition, the dynamic-mechanical associated with the composite viscoelastic behavior allows, as a function of temperature, the determination of important parameter such as the viscous stiffness, internal friction, damping factor, and glass transition temperature [12].

**Figure 1:**
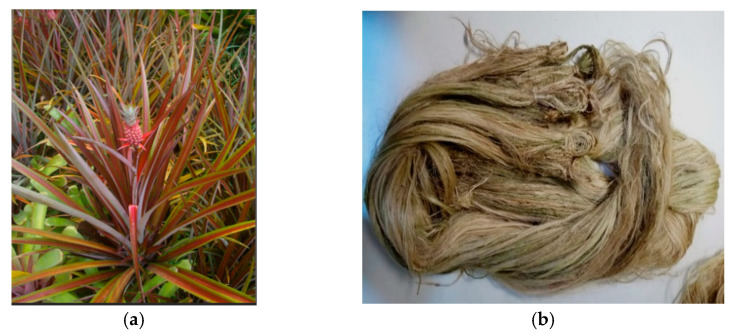
Curaua (*Ananas Erectifolius*): (**a**) plant and (**b**) bundle of fibers.

The curaua fiber (CF), one of the strongest natural fibers [21], extracted from the leaves of an Amazonian plant *Ananas erectifolius*, illustrated in Figure 1, has recently been GO-functionalized to reinforce epoxy matrix (EM) composites [13,16]. Raman and tensile tests were carried out in EM composites reinforced with 0.5 wt.% GO-functionalized 30 vol% CF, which showed an increase of ~40% tensile strength and ~60% Young’s modulus compared to the CF/M composite without GO functionalization [13].

**Figure 2:**
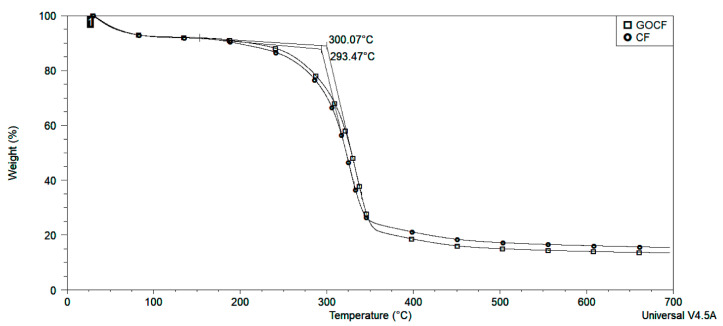
Thermogravimetric curves of CF and GOCF, reproduced with permission from [16].

Fourier transform infrared spectroscopy (FTIR), thermogravimetric analysis (TGA) pullout, and ballistic tests were also conducted in similar GO-30 vol% CF/EM [16]. In particular, TGA curves of the GO-functionalized curaua fiber (GOCF) displayed an increase in the thermal stability, which is shown in Figure 2 and reproduced from [16].

The results in [16] disclosed the appearance of FTIR bands characteristic of the molecular structure of GO despite its very low amount. Pullout tests revealed more than 50% higher interfacial shear strength for GOCF embedded in EM compared to non-functionalized CF in EM. Thermal degradation was retarded by the action of GO causing an insulation effect. Ballistic tests of GOCF/EM showed a performance comparable to that of Kevlar® as well as better armor physical integrity than the composite reinforced CF without GO functionalization [16].

Despite the aforementioned favorable properties, complementary tests are still required to assess the potential of GO-functionalized CF reinforced composites aiming to replace conventional synthetic fiber composites such as fiberglass in industrial applications. Thus, this brief report presents DMA results in 0.1 wt.% GO-functionalized 20 and 50 vol% CF reinforced EM composites. The objective was not only to investigate a lower and higher CF content than the previously 30 vol% CF composite [13,16], but also for the first time to unveil the viscoelastic behavior of these novel composites.

## 2. Materials and Methods

### 2.1. Materials

As supplied by the Federal University of Para, Belem, Brazil, the bundle of curaua fiber shown in Figure 1b was cleaned in running water and dried on a stove at 60 °C for 24 h. Cleaned and dried curaua fibers (CF) were then separated and cut to 150 mm.

The epoxy resin used as composite matrix was a diglycidyl ether of the bisphenol A (DGEBA) hardened with the catalyst triethylene tetramine (TETA) in the stoichiometric proportion phr 13. Both DGEBA and TETA were fabricated by Dow Chemicals, São Paulo and distributed by Epoxyfiber, Rio de Janeiro—RJ, Brazil.

The graphene oxide (GO) was produced in our laboratory at the Military Institute of Engineering (IME) by the modified Hummers and Offeman method [22], as described elsewhere [5]. In short, graphite was exfoliated and oxidated using a mixture of KMnO_4_ and a 9H_2_SO_4_/1H_3_PO_4_, which produced GO after ultracentrifugation.

### 2.2. Graphene Oxide (GO) Functionalization of Curaua Fibers (CFs)

The CFs were immersed in a 0.56 mg/mL of GO solution and maintained under constant agitation for 30 min in a mechanical shaker to optimize the contact of the GO, forming a nanometric thick coating onto the CF surface. Afterward, the CF soaked with GO film (GOCF) was placed on a stove at 80 °C for 24 h. The existence of GO nanofilm covering the CF surface was identified by Raman spectroscopy and scanning electron microscopy (SEM) [13].

### 2.3. Fabrication of Curaua Fiber-Epoxy Matrix Composites

Epoxy matrix (EM) composites with 20 and 50 vol% of GOCF/EM as well as non-functionalized CF/EM composites for the control were fabricated at IME, Rio de Janeiro-RJ, by compression molding. In a steel mold with dimensions of 150 × 120 × 11.9 mm, continuous and aligned fibers were hand-laid up in corresponding amounts. Still fluid DGEBA/TETA epoxy was then poured on the fibers. After closing the mold’s lid, a load of 5 tons was applied to the molds in a Skay hydraulic press at IME, Rio de Janeiro-RJ, during 24 h at room temperature. The corresponding volume fractions of fiber and resin were calculated by considering the density of CF as 0.92 g/cm^3^ and DEGBA/TETA as 1.11 g/cm^3^ [13].

### 2.4. Dynamic-Mechanical Analysis (DMA)

Standard DMA tests as per ASTM D7028-7 [23] were carried out using model Q800 TA Instruments equipment operating at a frequency of 1 Hz in a temperature range from 30 to 190 °C with a heating rate of 3 °C/min under a nitrogen atmosphere. Curves of storage modulus (E′), loss modulus (E″), and tangent delta (tan δ) were recorded usinga prismatic 50 × 12 × 3 mm specimen in the equipment’s three-point bend test mode.

### 2.5. Specimens Nomenclature

Table 1 presents the DMA specimen’s nomenclature following that previously adopted in [13,16].

### 2.6. Scanning Electron Microscopy (SEM)

SEM images of the GO-curaua fiber epoxy composites were analyzed in a model Quanta FEG250 FEI microscope thermofisher scientific, Hillsboro, OR, USA operating with secondary electrons at 10 kV. Samples were gold-sputtered for electron conduction.

## 3. Results and Discussions

### 3.1. Storage Modulus (E′)

**Figure 3:**
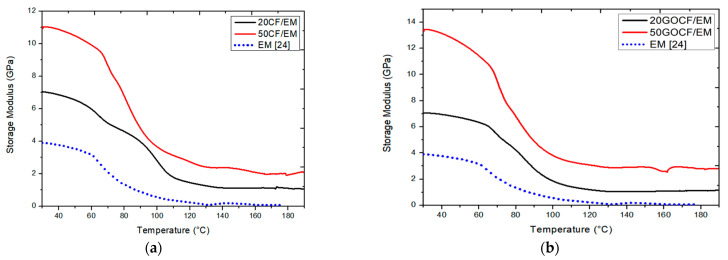
DMA storage modulus (E′) curves for epoxy composites incorporated with different amounts of (**a**) non-functionalized curaua fibers and (**b**) GO-functionalized curaua fibers. Neat epoxy reported in [24].

Figure 3 shows the variation of E′ with temperature for the investigated composites including that of neat epoxy from a previous work [24]. For both DMA results of E′, non-functionalized composites in Figure 3a, and GO-functionalized composites in Figure 3b, the values decreased with temperature but staying above the matrix (EM). This is a clear indication that, regardless of GO functionalization or not, the reinforcement with CF improved the viscoelastic stiffness of the composite at any level of temperature from 30 to 190 °C. Moreover, the values of the composites with 50 vol% of CF, either GO-functionalized in Figure 3b or not in Figure 3a, were markedly higher than the corresponding values for composites with 20 vol% of CF.

A comparison between the results in Figure 3a,b revealed that reinforcement with 20 vol% CF displayed similar values of E′ for both non-functionalized (20CF/EM) and GO-functionalized (20GOCF/EM) composites, along the investigated interval of temperature. In contrast, a significant increase in E′ was observed for the composite with 50 vol% of GO-functionalized (50GOCF/EM) compared to non-functionalized (50CF/EM) in Figure 3a,b, respectively. This might indicate that only for relatively higher levels of CF reinforcement such as 50 vol%, the viscoelastic stiffness could be affected by the GO functionalization. Such finding needs to be further investigated for a possible threshold in the amount of CF to be GO-functionalized. 

Another point of relevance is the sudden decrease in the value of E′, which occurs around 60–70 °C for all curves in Figure 3. This corresponds to the end of the glassy region for highly cross-linked thermoset polymer [25] such as the DGEBA/TETA matrix in the present work. In principle, this end of the glassy region might be associated with the transition to a rubbery amorphous region, which is a characteristic of the polymer matrix and is apparently not affected by the amount of CF or the GO functionalization in Figure 3.

### 3.2. Loss Modulus (E″)

**Figure 4:**
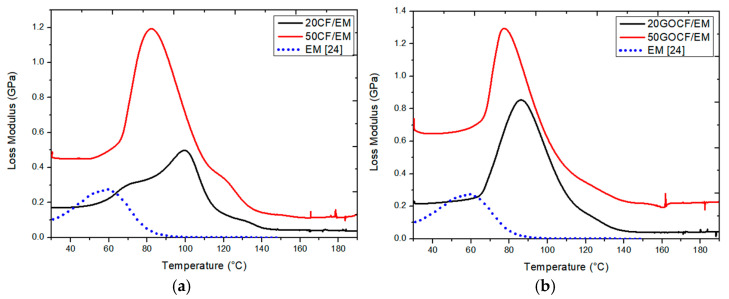
DMA loss modulus (E″) curves for epoxy composites with different amounts of (**a**) non-functionalized curaua fibers and (**b**) GO-functionalized curaua fibers. Neat epoxy reported in [24].

Figure 4 shows the variation of E″ with temperature for the investigated composites including that of the same neat DGEBA/TETA epoxy from a previous work [24]. In this figure, it is worth noting that for both epoxy composites with non-functionalized CF (Figure 4a) and GO-functionalized CF (Figure 4b), the values of E″ were above those of the neat epoxy, except for a limited temperature interval around 60 °C in which the E″ peak of neat epoxy rose slightly above the E″ values for 20CF/EM and 20GOCF/EM. Indeed, E″ is a viscous response of the material and considered as its tendency to dissipate the applied mechanical energy [25]. In the case of natural fiber composites, E″ is often described as the internal friction and should be higher, the greater the volume fraction of reinforcing fiber [12]. This would result in more fiber/polymer matrix interfacial surface. According to Mohanty et al. [26], the polymer molecular motion due to structural heterogeneities such as the natural fiber interface would directly increase the internal friction and, consequently, the value of E″.

Not only did the value of E″ change with the incorporation of a natural fiber, like the CF, but so did the amplitude of internal friction peaks in Figure 4. Moreover, the E″ peaks shifted to higher temperatures in relation to the epoxy matrix (EM) peak. In fact, the E″ peaks were associated with the onset of glass transition temperature, Tg, where a polymer changes from a glassy to rubbery state with total loss of crystallinity [27]. This transition to an amorphous structure is retarded by the incorporation of CF fibers, which hinders the movement of the DGEBA/TETA epoxy macromolecule chains toward a complete disordered arrangement. This is especially the case of non-functionalized composites (Figure 4a), in which the EM peak at ~60 °C shifted to ~100 °C for 20CF/EM and to ~85 °C for 50CF/EM. However, a rather shorter shift occurred for the GO-functionalized composites (Figure 4b), in which the EM peaks at ~60 °C shifted to ~85 °C for 20GOCF/EM and ~75 °C for 50GOCF/EM. It is herein speculated that the better adhesion provided by the GO functionalization to the CF prevents defects such as voids and microcracks to be generated during the DMA loading. Defects might make the chain motion difficult as apparently happened in the case of the non-functionalized CF composites in Figure 4a.

### 3.3. Tangent Delta (tan δ)

**Figure 5:**
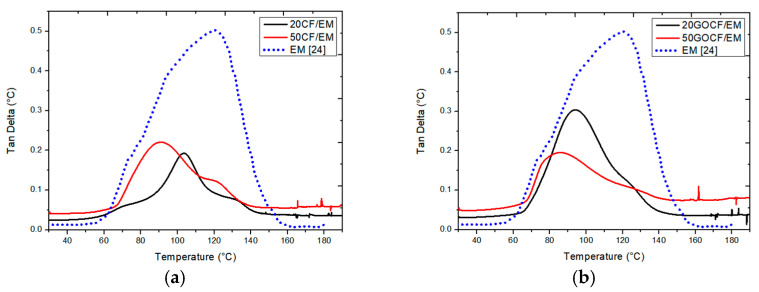
DMA tangent delta (tan δ) curves for epoxy composites incorporated with different amounts of (**a**) non-functionalized curaua fibers and (**b**) GO-functionalized curaua fibers. Neat epoxy reported in [24].

Figure 5 shows the variation of tan δ with temperature for the investigated composites including that of the same neat DGEBA/TETA epoxy from a previous work [24]. The tangent delta is the ratio between the DMA loss and storage modulus (tan δ = E″/E′) and, as such, a dimensionless number associated with the mechanical damping of the material. According to Saba et al. [25], a high value of tan δ is indicative of a material having a great non-elastic strain component upon load while a low tan δ value indicates higher elasticity. The temperature at the peak of tan δ is considered the dynamic T_g_.

The tan δ curves in Figure 5 show that for both epoxy composites with non-functionalized CF (Figure 5a) and GO-functionalized CF (Figure 5b), there are relatively smaller peaks at lower temperatures compared to the neat epoxy. In fact, the incorporation of natural fiber in a polymer matrix such as the CF into EM in the present work reduced the mobility of the polymer molecule chains at the fiber/matrix interface. This contributes to lower the mechanical energy loss by the imposed load in relation to its storage capacity [25]. Since the mechanical damping in a polymer is a consequence of molecular movement, the reinforcement of EM with CF or GOCF caused a significant decrease in the damping factor from ~0.5 to less than 0.3. 

Moreover, the temperature at the tan δ peak might also be understood as an upper limit for dynamic T_g_, at which the whole polymer structure becomes amorphous. This may also be visualized as the end of the sudden decrease in the E′ curve (Figure 3), finishing the structural transition from glassy to rubbery. For the neat epoxy, this dynamic T_g_ took place at ~120 °C, while for the investigated composites in Figure 4, the tan δ peak temperatures were reduced to ~50–100 °C. However, no apparent effect of GO functionalization exists regarding the total amorphization of EM.

### 3.4. Qualitative Discussion

Table 2 presents the main parameters obtained from the E′, E″, and tan δ curves in Figure 2, Figure 3 and Figure 4, respectively.

The results in this table reveal a significant effect of both GO functionalization and amount of fiber on important DMA parameters of CF reinforced epoxy composites. With GO-functionalized 50 vol% CF, the composite storage modulus was improved by more than 250% with respect to the neat epoxy EM as well as more than 20% compared to non-functionalized 50CF/EM and more than 90% compared to the 20GOCF/EM. As for the loss modulus, the 50GOCF/EM composite was enhanced by more than 600% as compared to EM as well as more than 40% to the non-functionalized 50CF/EM and 200% compared to the 20GOCF/EM. 

The maximum internal friction associated with the peak in E″ was substantially affected by the amount of CF reinforcement, but not much by the GO functionalization. Indeed, the internal friction of either 50CF/EM and 50GOCF/EM in Table 2 was more than 300% greater that of EM and more than 30% greater than those of 20CF/EM and 20GOCF/EM. The reason for these changes in E′ and E″ can be attributed to the viscoelastic stiffness reinforcement and restriction caused by a natural fiber to polymer chain mobility, respectively [25,26,27].

In contrast, the maximum damping associated with peak in tan δ and the dynamic T_g_ temperature at the peak, decreased with the amount of CF and GO functionalization. As discussed, the reduction in polymer macromolecular chain mobility by both conditions impairs not only the composite damping capacity, but also contributes to its amorphization.

The transition temperature from the glassy to the rubbery conditions in Table 2 are slightly increased by the incorporation of CF but not affected by its GO functionalization. This suggests that a relatively very low amount, 0.1 wt.%, of GO existing in the composites does not practically interfere in the thermally activated mechanisms that promote the glassy to rubbery transition.

### 3.5. Scanning Electron Microscopy (SEM)

**Figure 6:**
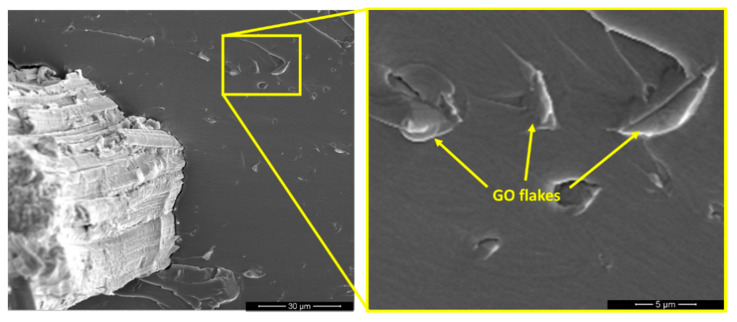
GO interface with epoxy matrix in curaua fiber composites.

Figure 6 shows the SEM image of GO flakes on the EM. The inset in this figure reveals that the GO/epoxy interface occurred at voids (yellow arrows) existing in the matrix. On the other hand, a rather uniform GO coating covered the surface of the curaua fiber.

### 3.6. The Cole–Cole Plot

The Cole–Cole plot is a representation of the loss modulus dependence with the storage modulus in a E″ versus E′ graph. This graph has been widely used to evaluate changes in viscoelastic properties [28]. In the case of fiber reinforced composites, the Cole–Cole plot is typically used to study structural changes that might occur in crosslinked polymers after the addition of fibers to the matrix [29]. In principle, homogeneous systems would be presented by a perfect semicircular graph [30] while heterogeneous systems would be shown as distorted graphs. 

**Figure 7:**
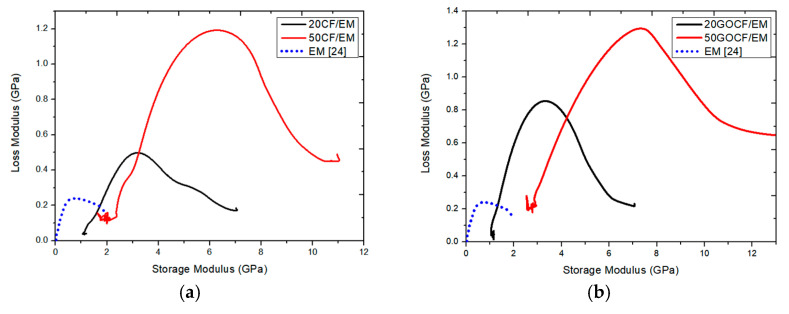
Cole–Cole plot of neat epoxy and epoxy composites: (**a**) 20CF/EM and 50CF/EM; (**b**) 20GOCF/EM and 50GOCF/EM.

Figure 7 shows the Cole–Cole plots for the neat epoxy as well as for the epoxy composites, both GO-functionalized and non-functionalized, reinforced with either 20 or 50 vol% CF. None of these graphs is a perfect semicircle. However, the neat epoxy has a better semicircular shape than the more asymmetric composites. It is worth observing that the 50GOCF/EM composite had the most distorted graph, which might indicate that both GO functionalization and amount of CF are related to more heterogeneous systems with impaired epoxy cross-linked bonding [31].

## 4. Summary and Conclusions

- Dynamic mechanical analyses (DMA) was conducted in both graphene oxide (GO)-functionalized and non-functionalized 20 and 50 vol% curaua fiber (CF) reinforced epoxy matrix (EM) composites. This brief report not only complements other reported properties but also extends the amount of CF in recent investigated EM composites.
- The DMA storage modulus (E′) of 50GOCF/EM was significantly enhanced by more than 250% with respect to the neat EM and more than 90% to 20 GOCF/EM. In the E′ variation with temperature, the transition from glassy to rubbery conditions was slightly increased by the amount of CF, but not affected by GO functionalization.
- The DMA loss modulus (E″) of 50GOCF/EM was enhanced by more than 600% compared to neat EM and 200% to 20GOCF/EM. The E″ peak associated with maximum internal friction was substantially enhanced by more than 300% with respect to the neat EM and by 30% to the 20GOCF/EM.
- The enhancement obtained in E′ and E″ can be attributed to the reinforcement in the viscoelastic stiffness caused by the addition of CF and restriction in EM chain mobility, respectively.
- The damping factor associated with the peak in tangent delta (tan δ) was reduced with the amount of CF and GO functionalization due to the reduction caused to the EM macromolecular chain mobility. Total EM amorphization related to dynamic glass transition temperature (T_g_) was impaired by CF addition and GO functionalization.
- Cole–Cole plots of E″ versus E′ revealed an increased distortion from a perfect semicircular graph, which would be associated with a homogeneous cross-linked structural system, with GO-functionalized CF addition to EM. This was assigned to a more heterogeneous structure with impaired epoxy cross-linked bonding.

## Figures and Tables

**Table 1:** Nomenclature adopted for DMA specimens.

Specimen	Nomenclature
Plain Epoxy	EM
Non-Functionalized 20 vol% Curaua Fiber Reinforced Epoxy matrix composite	20CF/EM
Graphene Oxide-Functionalized 20 vol% Curaua Fiber Reinforced Epoxy Matrix Composite	20 GOCF/EM
Non-Functionalized 50 vol% Curaua Fiber Reinforced Epoxy matrix composite	50CF/EM
Graphene Oxide-Functionalized 50 vol% Curaua Fiber Reinforced Epoxy Matrix Composite	50GOCF/EM

**Table 2:** DMA parameters for epoxy composites with 20 and 50 vol% of both non-functionalized and GO-functionalized curaua fibers. Neat epoxy DMA parameter obtained from [24].

DMA Parameter	EM	20CF/EM	20GOCF/EM	50CF/EM	50GOCF/EM
E′ at RT (GPa)	3.86	7.05	7.08	11.04	13.44
End of Glassy condition (°C)	59	65	61	68	64
Onset of Rubbery condition (°C)	84	96	95	99	96
E″ at RT (GPa)	0.09	0.17	0.22	0.45	0.67
Maximum Internal friction (GPa)	0.27	0.50	0.85	1.19	1.30
Begin glass transition T_g_ (°C)	59	99	82	82	77
Maximum Damping (Dimensionless)	0.51	0.19	0.22	0.31	0.20
Dynamic T_g_	121	101	102	95	100

## Data Availability

Not applicable.
